# Effects of Combined Gentamicin and Furosemide Treatment on Cochlear Macrophages

**DOI:** 10.3390/ijms23137343

**Published:** 2022-07-01

**Authors:** Liana Sargsyan, Austin R. Swisher, Alisa P. Hetrick, Hongzhe Li

**Affiliations:** 1 Research Service, VA Loma Linda Healthcare System, Loma Linda, CA 92357, USA; liana.sargsyan@va.gov (L.S.); swisheraustin@gmail.com (A.R.S.); alisa.hetrick@va.gov (A.P.H.); 2 Department of Otolaryngology-Head and Neck Surgery, Loma Linda University Health, Loma Linda, CA 92354, USA

**Keywords:** aminoglycosides, loop diuretics, cochlea, hair cells, ribbon synapses, macrophages

## Abstract

Combining aminoglycosides and loop diuretics often serves as an effective ototoxic approach to deafen experimental animals. The treatment results in rapid hair cell loss with extended macrophage presence in the cochlea, creating a sterile inflammatory environment. Although the early recruitment of macrophages is typically neuroprotective, the delay in the resolution of macrophage activity can be a complication if the damaged cochlea is used as a model to study subsequent therapeutic strategies. Here, we applied a high dose combination of systemic gentamicin and furosemide in *C57 BL/6* and *CBA/CaJ* mice and studied the ototoxic consequences in the cochlea, including hair cell survival, ribbon synaptic integrity, and macrophage activation up to 15-day posttreatment. The activity of macrophages in the basilar membrane was correlated to the severity of cochlear damage, particularly the hair cell damage. Comparatively, *C57 BL/6* cochleae were more vulnerable to the ototoxic challenge with escalated macrophage activation. In addition, the ribbon synaptic deterioration was disproportionately limited when compared to the degree of outer hair cell loss in *CBA/CaJ* mice. The innate and differential otoprotection in *CBA/CaJ* mice appears to be associated with the rapid activation of cochlear macrophages and a certain level of synaptogenesis after the combined gentamicin and furosemide treatment.

## 1. Introduction

Aminoglycosides are a group of potent, broad-spectrum antibiotics that are clinically prescribed to treat various aerobic bacterial infections, as well as unique pathologies such as multidrug-resistant tuberculosis [1]. The side effects of aminoglycosides include ototoxicity and nephrotoxicity. This raises concern when clinicians prescribe them more aggressively, such as in the case of cystic fibrosis patients [2]. The ototoxic risk can be further elevated in either local [3] or systemic [4] infections. In the biomedical research setting, scientists have been utilizing the ototoxic properties of aminoglycosides to deafen research animals and subsequently investigate candidate therapeutic strategies to cure hearing loss, including regenerative [5,6,7] and prosthetic [8,9] approaches.

Combining loop diuretics with aminoglycoside treatment is an effective approach to elicit immediate ototoxic damage [10]. In this accelerated version of a mouse ototoxicity model, the addition of loop diuretics (e.g., furosemide) disrupts the blood labyrinth barrier, which is similar to the blood brain barrier, and greatly elevates the concentration of aminoglycosides (e.g., gentamicin) in the cochlea [10,11]. We previously experimented with numerous doses in combinations of gentamicin and furosemide (G/F) in *C57 BL/6* mice (*B6*) and effectively induced cochlear damage to various degrees [12]. Using immunofluorescence techniques, this study demonstrated that combined G/F treatment did not decrease the synaptic ribbon density in the inner hair cell (IHC) until overt damage occurred to the outer hair cell (OHC). This observation was consistent with previous knowledge that the primary ototoxic target of aminoglycosides is the sensory hair cells of the mammalian cochlea, while the damage to the spiral ganglion neuron (SGN) is secondary [13,14]. In addition to the aforementioned ototoxic deafening demand, our study suggested that with appropriately titrated G/F dosing, sensory hair cells could be completely erased with a well-preserved neuronal compartment, creating a preferred pathological condition for prosthetic cochlear implantation.

Although a G/F protocol could promptly induce cochlear damage, the succeeding physiology is not necessarily stabilized right away. Increased cochlear macrophage activities have been reported to last months after a brief exposure to a moderate level of noise [15]. This finding has also been reported with multiday aminoglycoside treatment [16,17]. To our knowledge, the response of cochlear macrophages in the pathological tissue to G/F treatment has not been investigated in detail. Given the sophisticated behaviors of stress-activated macrophages, understanding this response is essential to determine the appropriate time frame to initiate therapy after ototoxic deafening. In general, damage-associated molecular patterns (DAMPs) produced by injured tissue can be recognized by pattern recognition receptors (PRRs) expressed on resident macrophages, and subsequently activates these innate immune cells. Activated cochlear macrophages secrete pro-inflammatory cytokines, resulting in deleterious effects and infiltration of extra immune cells [18]. On the other hand, Kaur et al. [19,20] suggested that the recruitment of macrophages can also promote the survival of SGNs and the spontaneous recovery of ribbon synapses upon cochlear injury. This is consistent with our previous study [12] which revealed prompt and evident synaptogenesis with G/F challenge when hair cells were well preserved.

It has been established that inflammation plays a major role in the ear pathology caused by noise overexposure [21,22,23]. Cochlear inflammation that occurs after acoustic or ototoxic drug exposure is considered sterile inflammation that manifests with elevated cytokine and chemokine levels and infiltrated leukocytes [21,24]. The overall course of the inflammatory response, from its initiation to its resolution, if completed swiftly, is beneficial and protects the host from infection and the risk of further tissue injury [25,26]. Alternatively, blunt anti-inflammatory approaches, such as the application of corticosteroids do present a certain protective effect on reducing the risk of hearing loss [27,28,29]. Here, we hypothesize that G/F treatment activates cochlear macrophages and triggers an episode of cochlear inflammation. Since the inflammatory response is associated with both pathology and protection, we will attempt to elucidate the interactions between cellular damage and immunoprotective events within the cochlea.

Given that a large inter-animal ototoxic response was observed after the G/F treatment [12], a fixed dose of G/F combination is practically selected in the present study, thus permitting our focus to be on other experimental parameters as well as the activation of cochlear macrophages. Thus, the aims of this study were to (1) characterize hair cell survival and synaptic density at multiple frequency locations, instead of limiting only to the 12-kHz location; (2) extend the morphological assessment to 15-day posttreatment; (3) as a comparative approach, in addition to *B6* mice that exhibit age-related hearing loss phenotype, study *CBA/CaJ* mice (*CBA*) that do not; and most importantly, (4) investigate macrophage activity in the organ of Corti in both wildtype strains, in an attempt to associate the G/F-activated macrophages with other physiological and morphological alterations following ototoxic challenge.

## 2. Methods

### 2.1. Mice and Gentamicin/Furosemide (G/F) Treatment

*C57 BL/6* (JAX stock #0664) and *CBA/CaJ* mice (JAX stock #0654) of both sexes at the age of 6–8 weeks were recruited and bilateral data from 29 *B6* mice and 32 *CBA* mice were included in the study. Animals were housed in a Specific Pathogen Free-modified room. Experimental animals were injected intraperitoneally (*i.p.*) with a single dose of 400 mg/kg *b.w.* gentamicin (Sigma-Aldrich, Sigma-Aldrich, St. Louis, MO, USA, G1264, Lot #SLBG7734 V) followed after 30 min by 200 mg/kg *b.w.* furosemide (Fresenius Kabi, Germany), abbreviated to 400 G/200 F. This dosing combination was selected because it effectively results in cochlear injury in *B6* mice with a low mortality rate according to our previous research [12]. Mice typically underwent auditory brainstem response (ABR) tests prior to G/F treatment and immediately before tissue harvest at either 48 h or 15-day posttreatment. All animal work was carried out using protocols approved by the Institutional Animal Care and Use Committee of the Jerry L. Pettis Memorial VA Medical Center, Loma Linda, CA, USA. Animal use procedures conform with federal regulations regarding personnel, supervision, record keeping, and veterinary care.

### 2.2. ABR Measurement

Individual anesthetized mice (ketamine 65 mg/kg/xylazine 13 mg/kg, *i.p.*) were stimulated with a closed tube sound-delivery system sealed into the outer ear canal. ABRs, using tone-burst stimuli (5-ms duration, 1-ms rise/fall times, n = 512 repeats) at 4, 8, 12, 16, 24, and 32 kHz, with 5-dB steps, ranging from 0 to 90 dB SPL, were recorded using a Tucker-Davis Technologies (TDT) System 3 (Alachua, FL, USA). Thresholds were determined as the lowest signal intensity at which a response could be identified, and wave I peak-to-peak amplitudes were measured from averaged response waveforms at each sound level.

### 2.3. Tissue-Processing, Image Acquisition and Processing

Cochlear samples were immersion-fixed overnight in 4% paraformaldehyde (pH 7.4), followed by EDTA (10%, pH 7.4) decalcification at room temperature (RT). To prepare the cochlear whole-mounts, the membranous labyrinth of the cochlea was micro-dissected under a dissecting microscope to remove the softened otic capsule, stria vascularis, Reissner’s membrane, and tectorial membrane. After further immersion-fixation in 4% paraformaldehyde, specimens were permeabilized in 1% Triton-X solution for 1 h at RT. Specimens were then incubated at 37 °C overnight with primary antibodies including mouse monoclonal anti-CtBP2 IgG1 (612044, BD Biosciences, 1:200), rabbit monoclonal anti-Iba1 IgG (Cat# ab178846, Abcam, Cambridge, UK; 1:200), rabbit polyclonal anti-Myo7 a IgG (PA-936, Thermo Fisher, 1:200), and/or mouse monoclonal anti-GluR2 IgG2 a (MAB397; Millipore, Burlington, MA, USA; 1:1000). After rinsing, the specimens were incubated with the Alexa Fluor 568- and 647-conjugated secondary antibodies together with Alexa Fluor 488 phalloidin (1:1000) at 37 °C for 1 h in the dark. The modiolus was removed from the tissue after final washing, and the epithelia were divided into segments and mounted on slides with the anti-fade fluorescence mounting media VectaShield (Vector Labs, Burlingame, CA, USA) under uniform 60× magnification. Immunolabeled images were acquired using a laser confocal microscope (Fluoview FV3000, Olympus Corp., Center Valley, PA, USA).

The ribbon density (number of ribbons per IHC) and macrophage density (cells per 100 µm sensory epithelium) were quantified at multiple cochlear locations using ImageJ (NIH, Bethesda, MD, USA). At each location, ribbons from at least 30 IHCs were counted and averaged to determine the ribbon density, avoiding the need to define the cell boundary of each IHC. Data were analyzed using Prism (GraphPad Software, La Jolla, CA, USA) software. Statistical methods included Fisher’s exact test, two-way ANOVA with *Post hoc* Šídák’s multiple comparisons, and Pearson’s correlation with simple linear regression analysis. A *p*-value of <0.05 was considered statistically significant.

## 3. Results

Among the animals that received 400 G/200 F treatment, 2 out of 24 (8.3%) *B6* mice and 9 out of 26 (34.6%) *CBA* mice failed to survive after the injections, suggesting that the combined protocol generated a substantial level of systemic toxicity. A two-sided Fisher’s exact test indicated that *CBA* mice were more vulnerable to the high dose treatment (*p* = 0.0395). Data from the mice that survived and nontreated control animals are reported as follows.

### 3.1. G/F Treatment Induced More ABR Threshold Shift in C57 BL/6 Mice

**Figure 1:**
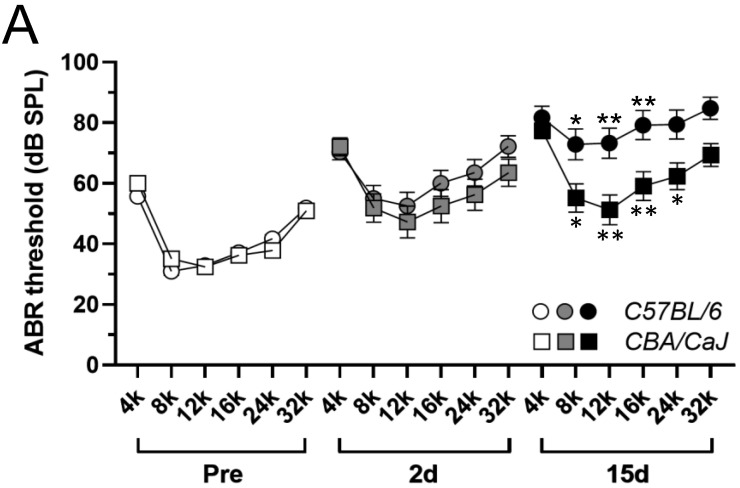

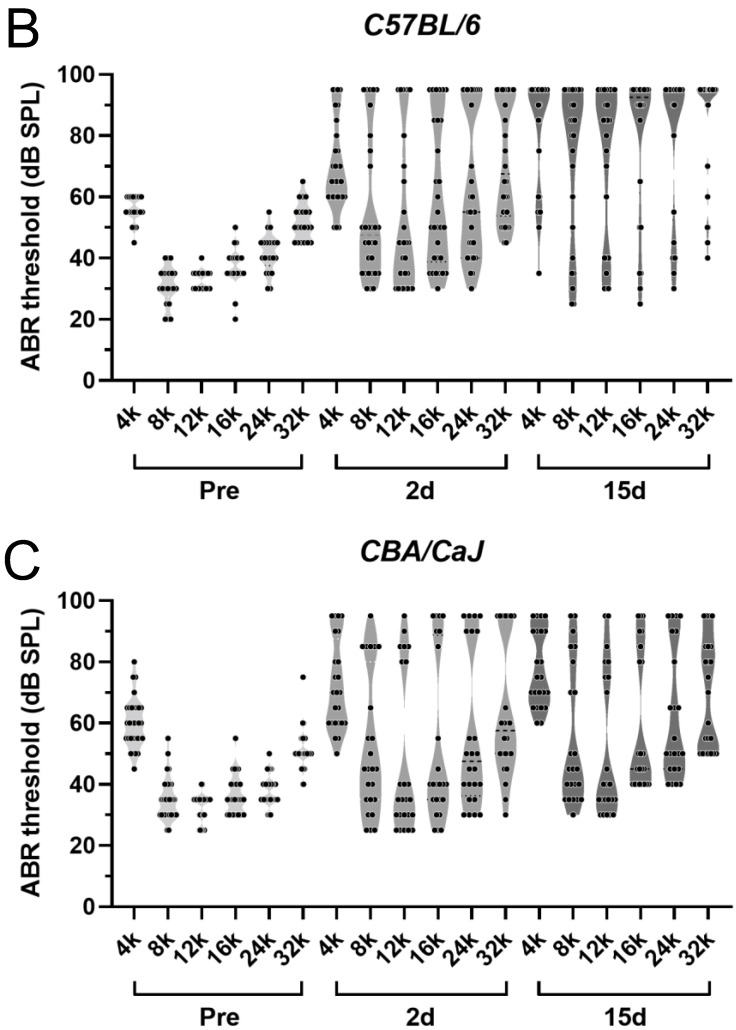
ABR thresholds elevated differentially in *B6* and *CBA* mice after 400 G/200 F treatment. (**A**) Average ABR thresholds before, 2 days after, and 15 days after the treatment, in *B6* (circles) and *CBA* (squares) mice. 5-pointed asterisks depict the statistical significance of ABR threshold comparisons between 15-d *B6* and 2-d *B6* mice, while 6-pointed asterisks depict the comparisons between 15-d *B6* and 15-d *CBA* mice. * *p* < 0.05, ** *p* < 0.01, *post hoc* Šídák’s multiple comparisons tests. Error bar = SEM. (**B**) Violin plots of ABR thresholds from individual *B6* mice. (**C**) Violin plots of ABR thresholds from individual *CBA* mice.

Prior to G/F treatment, baseline ABR audiograms from young adult *B6* and *CBA* mice were nearly superimposed upon each other (Figure 1A) and a two-way ANOVA test did not detect any threshold variation in their hearing sensitivities (Table 1). G/F treatment induced overt threshold elevation in both strains 2 days after the treatment, while the threshold shifts in *B6* mice were marginally higher, which were statistically significantly compared to those in *CBA* mice (*p =* 0.0462, see Table 1 for statistical details). Fifteen days after treatment, further threshold shifts in *B6* were evident (*p <* 0.001), and the shifts in *CBA* mice were marginal (*p =* 0.0453) compared to 2 days posttreatment. *Post hoc* Šídák’s multiple comparisons tests identified significant threshold elevation at individual frequencies of 8, 12, and 16 kHz in *B6* mice at 15-day posttreatment compared to 2 days posttreatment (5-pointed asterisks in Figure 1A), and significant elevation at 8, 12, 16, and 24 kHz compared to *CBA* mice at the same time point (6-pointed asterisks in Figure 1A).

Although a strain-dependent disparity in ABR thresholds was observed after G/F treatment, it should be noted that the ototoxic challenge induced great inter-animal variation, as reported earlier with other G/F dosing combinations [30]. A characteristic bipolar distribution of ABR thresholds could be seen at each tested frequency 2 days posttreatment, in either strain, when individual threshold values were presented in violin plots (Figure 1B,C). While the bipolar signature was largely maintained 15-day posttreatment in *CBA* mice (Figure 1C), the individual ABR threshold values shifted toward the upper pole in *B6* mice (Figure 1B). Suprathreshold ABR wave-I amplitudes were consistently reduced after the G/F treatment in both strains, with greater reduction observed 15-day posttreatment in *B6* mice. See Supplementary Figure S1 for representative wave-I amplitude distributions to 12-kHz tones at 90, 85, and, 80 dB SPL.

### 3.2. Morphological Analysis of ABR Correlates in the Cochlea

**Figure 2:**
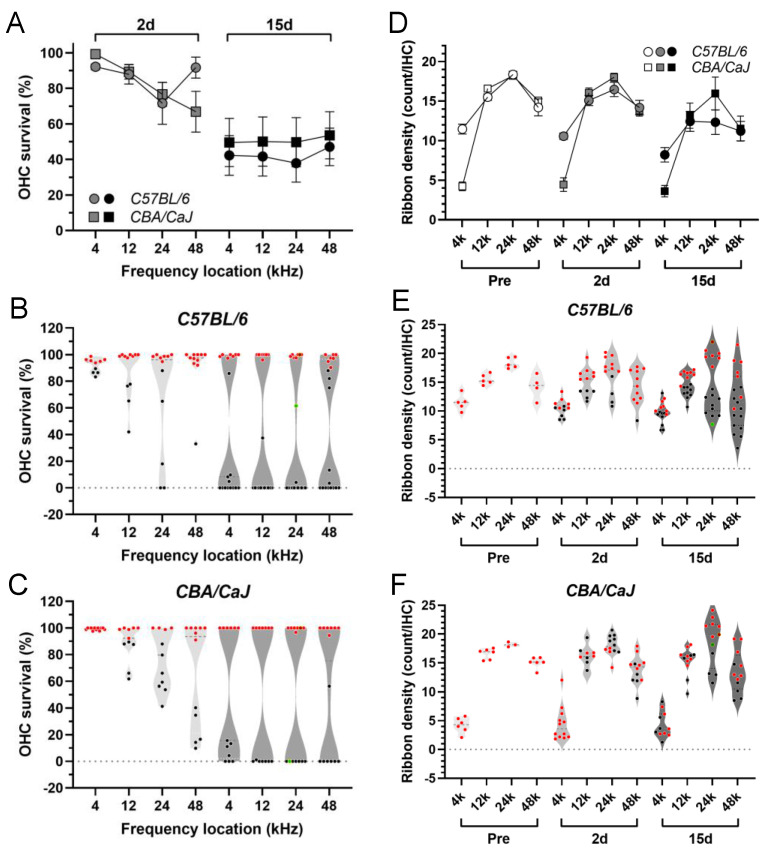
OHC survival and synaptic ribbon density after 400 G/200 F treatment. (**A**): Average percentages of OHC survival at multiple frequency locations 2 and 15 days after treatment in *B6* (circles) and *CBA* (squares) mice. Error bar = SEM. (**B**): Violin plots of OHC survival from individual *B6* mice. Red symbols indicate a survival rate ≥90%. (**C**): Violin plots of OHC survival from individual *CBA* mice. (**D**): Average ribbon density 2 and 15 days after the treatment in *B6* and *CBA* mice. Error bar = SEM. (**E**): Violin plots of ribbon density from individual *B6* mice. (**F**): Violin plots of ribbon density from individual *CBA* mice. Red symbols indicate the OHC survival rate ≥90% from the same cochlear sample analyzed.

The sensory component and the neural component of ABR correlates in the cochlea were examined using OHC survival and synaptic ribbon density as the corresponding morphological references. G/F challenge resulted in progressive OHC loss with great inter-animal variation (Figure 2A). The degree of OHC survival was comparable between *B6* and *CBA* mice at either posttreatment time point (*p =* 0.5901 for 2 d, *p =* 0.3260 for 15 d, two-way ANOVAs). Drastic bipolar distribution was seen in OHC survival 15-day posttreatment in both mouse strains (Figure 2B,C); i.e., the OHC loss was largely all-or-none.

The synaptic ribbons of the IHC were also affected by the G/F challenge. Progressive reduction of ribbon density was evident in *B6* mice after the G/F treatment (Figure 2D), see Table 2 for ANOVA-based statistics. In contrast, the mean of ribbon densities was comparably stable after the treatment in *CBA* mice. Only a marginal reduction was detected in the 15-day posttreatment group compared to the pre-treatment group (*p =* 0.0451, two-way ANOVA, Table 2). Note that there was a small number of cochlear samples that presented massive IHC loss in the 15-day posttreatment groups. These samples were excluded from the violin plots with individual ribbon densities (Figure 2E,F), including 3 out of 20 *B6* cochleae (15%) and 2 out of 14 *CBA* cochleae (14%). However, all cochlear samples were included in the aforementioned ANOVA-based group analysis. The Pearson correlation between ribbon density and the corresponding ABR wave-I amplitude at the highest tested sound level (90-dB SPL) was analyzed at the 12-kHz location from the available dataset (Supplementary Figure S2). The only significant correlation was found in *B6* mice at 2 days posttreatment.

To summarize broadly, G/F treatment caused a comparable scale of OHC damage between *B6* and *CBA* mice, but a differential stress potential on the ribbon density was indicated by the more progressive ribbon reduction in *B6* mice. Collectively, this suggested that the excessive ABR threshold elevation in *B6* mice was either due to the neural damage that was restricted at the SGN level, or was due to the loss of OHC function of amplification even when these OHCs morphologically survived.

### 3.3. Elevated Macrophage Activity after G/F Treatment

**Figure 3:**
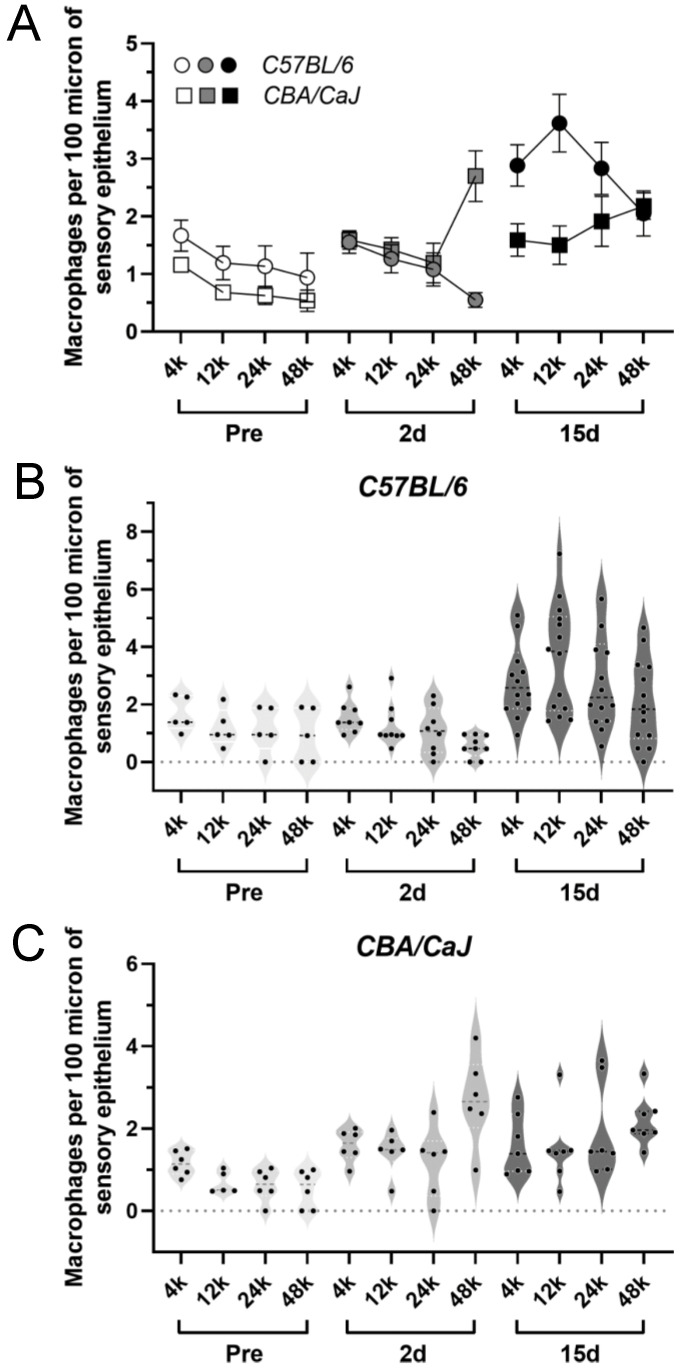
400 G/200 F treatment modified macrophage density differentially in the basilar membrane. (**A**): Average macrophage density at multiple frequency locations before, 2 days after, and 15 days after the treatment, in *B6* (circles) and *CBA* (squares) mice. Error bar = SEM. (**B**): Violin plots of macrophage density from individual *B6* mice. (**C**): Violin plots of macrophage density from individual *CBA* mice.

Cochlear macrophages in the basilar membrane (BM) were quantified by anti-Iba1 immunolabeling. Iba1-positive macrophages in the cochlear sensory epithelium and the medially located macrophages in the osseous spiral laminar (OSL), with identifiable processes towards the IHC synaptic zone, were included in the quantification. Prior to G/F treatment, cochlear BM macrophages were scarce, with a gradual downwards trend from the cochlear apex to the base. The baseline BM macrophage density was moderately lower in *CBA* mice compared to *B6* mice (Figure 3A, *p =* 0.0101, two-way ANOVA, Table 3). G/F challenge significantly elevated the macrophage density at 15-day posttreatment in both strains (Figure 3A, *p <* 0.0001, Table 3), while a consistently higher macrophage density was observed in *B6* mice *vs CBA* mice (Figure 3A–C, *p =* 0.0019). Intriguingly, the increase in macrophage density primarily occurred at the early phase after G/F treatment in *CBA* mice (*p <* 0.0001, pre- *vs* 2 d posttreatment) rather than the late phase (*p =* 0.7597, 2 d *vs* 15 d posttreatment), implying a potentially beneficial effect of preventing further ototoxic damage in the cochlea.

**Figure 4:**
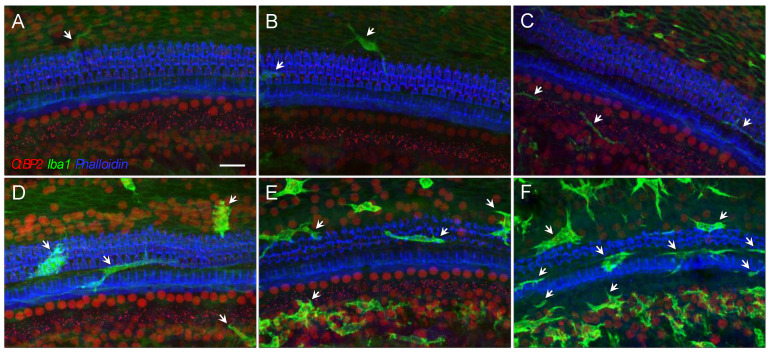
Morphological alteration at the apical-to-middle cochlear location after 400 G/200 F treatment. (**A**–**C**): Complete hair cell survival (blue phalloidin labeling), intact synaptic ribbons (red punctate), and macrophages (green, identified by white arrows) in quiescence were present in confocal z-projection images from individual *B6* control mice at the cochlear location corresponding to 12 kHz. Scale bar = 20 µm. Fifteen days after G/F treatment, the degree of hair cell survival varied, from complete OHC survival (**D**), complete OHC loss (**E**), complete hair cell loss including both OHCs and IHCs (**F**). These representative images demonstrate increased macrophage activity in all cases and compromised ribbon synaptic integrity when OHC loss occurred.

In addition to the quantified macrophage density, G/F-enhanced macrophage activity was reflected by the shape of the cells, as well as their immunofluorescent signal strength. Using cochlear samples at the frequency location equivalent of 12 kHz from *B6* mice as examples, prior to the ototoxic treatment (Figure 4A–C), the macrophages were typically in a quiescent state with long processes and weak fluorescent signals that could barely be recognized above the nonspecific signals exhibited by surrounding fibrocytes (Figure 4A). Fifteen days posttreatment, the sensory epithelium demonstrated various degrees of damage, ranging from no overt hair cell loss (Figure 4D), to complete OHC loss (Figure 4E), and to complete hair cell loss including both OHCs and IHCs (Figure 4F). In all scenarios, macrophages were classically activated to amoeba shapes with robust fluorescent signals, suggesting activation of the BM macrophage proceeds the death of sensory cells, but does not necessarily prohibit their death.

**Figure 5:**
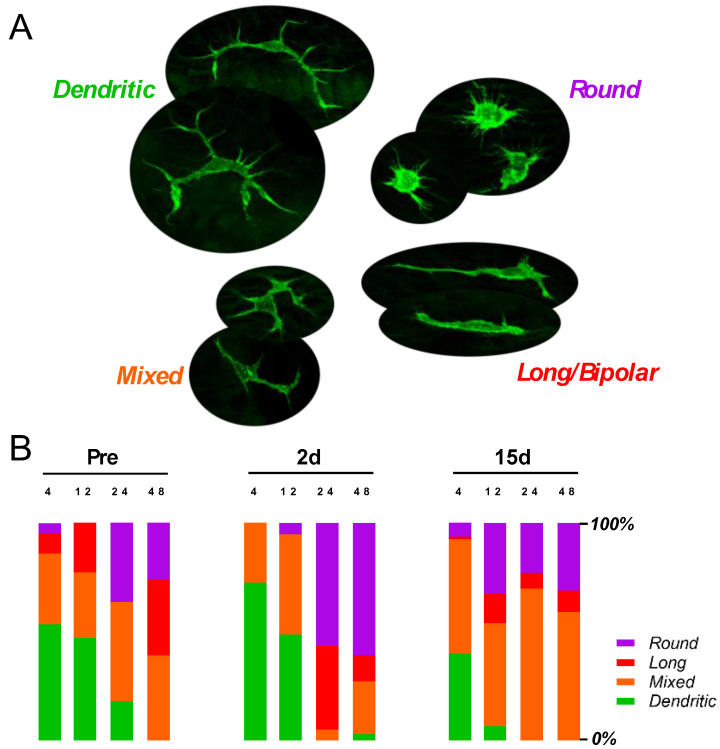
400 G/200 F treatment altered the composition of activated macrophages in the basilar membrane (**A**) Macrophages are morphologically categorized into quiescent dendritic shapes, activated round and long shapes, and mixed shapes that fall in between the extremities. (**B**) Partition of the BM macrophages at multiple cochlear locations before, 2 days after, and 15 days after treatment in *B6* mice. Note the composition analysis does not reflect the G/F-enhanced macrophage numbers.

To adequately summarize the morphological change with G/F treatment, we categorized macrophages into four types. The shapes include dendritic, mixed, long, and round. Dendritic shapes represented the quiescent macrophages with extended arborized processes; long/bipolar, and round represented activated macrophages with amoeboid shapes; mixed reflected shapes that fell in between the two extremities (Figure 5A). In the apical basilar membrane of non-treated *B6* mice, a substantial portion of macrophages were dendritic in shape, while round and long shapes dominated in the base. The distribution pattern in the two ends of the cochlea was largely preserved after the G/F treatment (Figure 5B). It should be noted that the number of macrophages did increase after the treatment. The round and long composition increased overtly in the middle region of the cochlea. A drastic increase was seen at the 24 kHz location 2 days posttreatment and at the 12-kHz location 15-day posttreatment.

### 3.4. Elevated Macrophage Activity Associated with Cochlear Damage in B6 Mice

**Figure 6:**
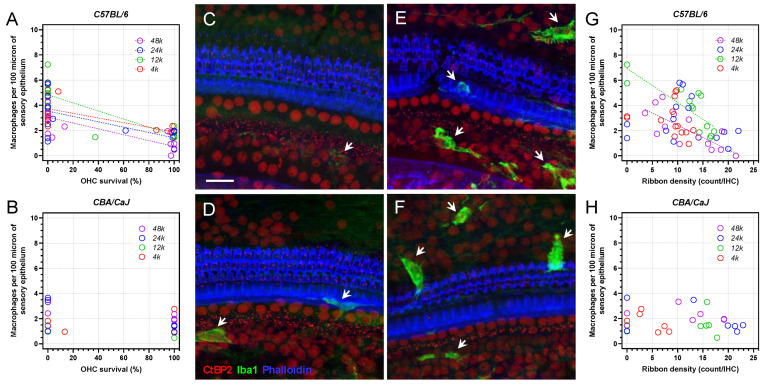
Macrophage density correlated to OHC survival and synaptic ribbon density in *B6* mice but not in *CBA* mice 15 days after G/F treatment. (**A**): Macrophage density and OHC survival were correlated at all examined cochlear locations 15-day posttreatment in *B6* mice, by Pearson’s r test. Such correlation was not observed in *CBA* mice (**B**). Confocal images demonstrate intact cochlear morphology of the sensory epithelium with moderated macrophage activities (while arrows) at the 24-kHz location 15 days after G/F treatment from a *B6* (**C**) and a *CBA* (**D**) mouse. Scale bar = 20 µm. When G/F-induced ototoxicity was evident, reflected by a partial or complete OHC loss, elevated macrophage activity could be seen from many individual samples at the 24-kHz location, e.g., from a *B6* (**E**) and a *CBA* (**F**) mouse. (**G**): Macrophage density and ribbon density were correlated at 12-kHz and 48-kHz frequency locations in *B6* mice. Such correlation was not observed in *CBA* mice (**H**).

With a more diversified macrophage density 15-day posttreatment, the density value from individual samples was reversely correlated with the polarized OHC survival in *B6* mice (Figure 6A) at all examined frequency locations. Such correlation was not observed in *CBA* mice (Figure 6B).

To demonstrate the correlation between macrophage activity and ribbon synaptic damage, we selected individual cochlear samples at the 24-kHz frequency locations with very high ribbon densities (brown symbols in Figure 2E,F) or low ribbon densities (green symbols) from both *B6* (Figure 6C,E) and *CBA* (Figure 6D,F) mice. The macrophage activity, represented by Iba1-immunofluorescent intensity and by the number of macrophages, appeared lower with high ribbon densities (Figure 6C,D). Conversely, the activity appeared higher with low ribbon densities (Figure 6E,F). In the pooled data, however, the reverse correlation between macrophage density and ribbon density was only observed at 12-kHz and 48-kHz frequency locations in the *B6* mice (Figure 6G), but not in *CBA* mice (Figure 6H).

No significant correlation was observed 2 days posttreatment between macrophage density and OHC survival, or between macrophage density and ribbon density, in either mouse strain (Supplementary Figure S3).

### 3.5. Postsynaptic AMPA Receptor Density

**Figure 7:**
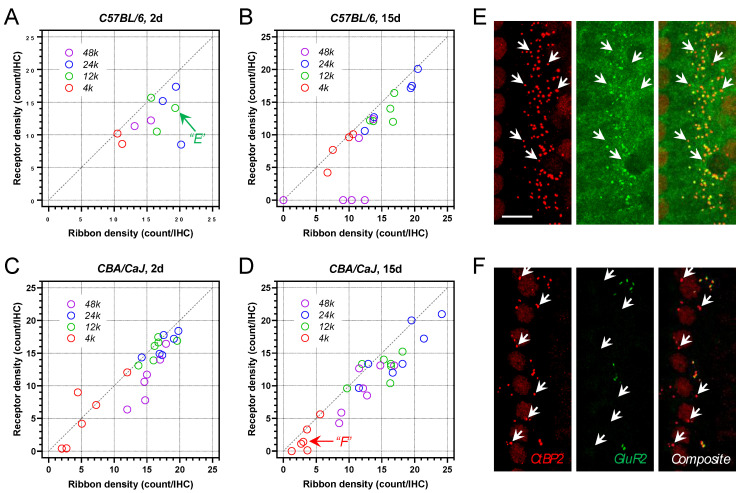
Correlation between presynaptic ribbon density and post-synaptic AMPA receptor density. (**A**) 2 days after 400 G/200 F treatment in *B6* mice. (**B**) 15-day posttreatment in *B6* mice. (**C**) 2-day posttreatment in *CBA* mice. (**D**) 15-day posttreatment in *CBA* mice. (**E**) Presynaptic ribbons (red) and post-synaptic AMPA receptors (green) at the 12-kHz location from a *B6* mouse 2-day posttreatment. Arrows indicate orphan ribbons. Scale bar = 10 µm. (**F**) Ribbons and receptors at the 4-Hz location from a *CBA* mouse 15-day posttreatment. Arrows indicate orphan ribbons.

In order to achieve reliable immunofluorescent labeling, up to three fluorescence channels were used in the present study. Thus, when Iba1 macrophages were identified in a sample, only presynaptic ribbons were co-labeled, as well as the referencing phalloidin labeling. In a subset of animals, postsynaptic AMPA receptors were identified by the anti-GluR2 immunolabeling with synaptic ribbons to confirm that the ribbon density would reasonably reflect the overall synaptic integrity. As demonstrated in Figure 7A–D, the receptor density was generally correlated with the ribbon density at all examined frequency locations. The receptor density could be lower than its presynaptic counterpart, resulting in identifiable orphan ribbons, which were evident in cochlear samples with complete OHC loss (Figure 7E,F). This finding suggested a certain level of susceptibility of AMPA receptors to G/F-treatment. Alternatively, the observation could suggest other postsynaptic receptors rather than AMPA receptors were potentially recruited to the synaptic zone after G/F treatment [31]. In summary, the ribbon density reported here could marginally overrepresent the overall synaptic integrity in the study.

## 4. Discussion

With a fixed G/F dose combination, we observed large inter-animal variation with overt bipolar data distribution in ABR thresholds, in ABR suprathreshold responses, and in OHC survival. We also confirmed that sensory hair cells are the primary ototoxic target in the cochlea. Consistent with previous reports [32], *CBA* mice, if they survive, are less vulnerable to aminoglycosides than *B6* mice in terms of gross cochleotoxicity, as gauged by all physiologic and morphologic assessments used in the present study.

Cochlear macrophages were rapidly activated by G/F treatment, and an association was observed between the OHC damage and macrophage activation. These activated macrophages might linger in the milieu after ototoxic insult and the clearance of damaged tissues, including dead hair cells. Proper manipulation of these macrophages, or associated innate immune responses, could alleviate the inner ear damage due to various environmental insults [12,30]. Conversely, these cells might also interfere with subsequent studies on candidate bioengineering interventions, either neuro-regeneration or cochlear prosthesis.

### 4.1. G/F Dosing Protocol and Cochlear Damage

The combination of 400 G/200 F was confirmed to be the one that led to severe cochlear damage in both mouse strains, similar to the 200 G/400 F dosing protocol reported previously [12]. DPOAE tests were not acquired in the present study, but based on the ABR measurement and the cochlear morphological analysis, OHC damage typically spread throughout the entire frequency range when it occurred, particularly in *B6* mice. Thus, when it is essential to restrict cochlear damage at the base and to preserve apical OHC survival, such as for a hybrid cochlear implant animal model [33], a dose combination at 200 G/200 F or lower is suggested, preferably by further reducing the dosage of furosemide.

Cochlear synaptic transmission is a critical determinant to the quality of auditory perception. It is affected primarily in age-related and noise-induced auditory dysfunction long before significant hearing loss is detected [34]. The main target in these otopathologies is the ribbon synapses at the base of inner hair cells. Certain evidence of synaptopathy has been presented with cisplatin-induced hearing loss [35] but it has yet to be clearly demonstrated in aminoglycoside-induced hearing loss [12]. Previously, we generated a broad spectrum of cochlear injuries based on various G/F dosing combinations, and synaptic damage that is independent of OHC injuries was not evident. Here, with a fixed G/F dosage, we surveyed ribbon synaptic conditions at multiple frequency locations with a longer posttreatment time frame. The reduction of ribbon density with intact OHC survival was unpopular in either *B6* or *CBA* mice (Figure 2E,F; Supplementary Figure S2). Interestingly, the reduction of ABR wave-I amplitude exceeded the deterioration in OHC and ribbon synaptic morphologies in *B6* mice (Supplementary Figure S1), which implies a certain degree of G/F-induced damage at the SGN level [20,36]. Nevertheless, the present study again confirms that G/F-induced ototoxicity hardly results in hidden hearing loss (i.e., the type of synaptopathy with restricted deficits in the auditory nerve fibers with low spontaneous rates).

### 4.2. Cochlear Inflammation and Macrophage Activation

Neuroinflammation is implicated in mammalian inner ear diseases including hearing loss and Meniere’s disease [22,37,38]. The activation of cochlear macrophages is the hallmark event of cochlear inflammation, occurring after a one-time treatment of aminoglycosides and loop diuretics and has previously been studied using kanamycin and furosemide [20]. To our knowledge, this is the first report on the cochlear macrophage with combined G/F treatment. One possible reason for the lack of research on gentamicin in this combined approach is due to the fact that when gentamicin is used alone in a multiday ototoxic protocol, mice typically experience systemic toxicity prior to ototoxicity. Several other studies have documented cochlear macrophage activation after multiday aminoglycoside treatment, including amikacin [16] and kanamycin [14,17].

Basilar membrane (BM) macrophages identified in the present study included the population of macrophages that resided in the organ of Corti and the OSL, as well as some macrophages in the Rosenthal’s canal with recognizable processes through the habenula perforate, but did not include the ones attached to the bottom of the BM within the Scala tympani due to the nature of the wholemount sample preparation. Macrophages were identified by their Iba1 activity and characterized by their shapes. Other frequently selected cell surface markers in the auditory peripherals include CD45 and F4/80, but are typically deemed to identify the same population of cochlear macrophages. In the present study, the baseline level of macrophage density was considerably low and many samples did not present any macrophage, which is inconsistent with previous literature [39,40]. Based on the technique used here, we cannot rule out the possibility that some dormant BM macrophages were actually located in the area but failed to be identified with Iba1 immunolabeling. We suspect the presence of these dormant macrophages because of the observation of weakly labeled Iba1+ macrophages in some control samples. The shape of BM macrophages varies along the length of the cochlea, from apically located dendritic shapes that occupy a large area to the basally located amoeba shapes which are considered to be more activated. The transition in the shapes occurred somewhere in the middle of the cochlea. G/F treatment pushed the site of this transition towards the cochlear apex, reminiscent of a similar observation in noise-induced BM macrophage activation [15].

BM macrophages actively responded to G/F-induced cochlear pathologies, proceeding the OHC loss and synaptic damage, if there was any. BM macrophage activation was associated with the severity of deterioration in hearing sensitivity and with the OHC loss, but less so with the synaptic density reduction. This observation supports the neuroprotective potential of the cochlear macrophages, particularly the OSL-located macrophages. Without G/F challenge, greater average baseline macrophage density was observed across the entire cochlea in *B6* mice (Table 3), implying the macrophage-dependent innate immune activity could play an essential role in the expediated age-related hearing loss in *B6* mice [37].

### 4.3. Differential Damage between CBA and B6

The mouse strain effect on the cochlear vulnerability to the G/F challenge was evaluated throughout the study. We found that the treatment induced a rapid recruitment of resident macrophages at the damaged region in *CBA* mice despite hair cell survival. This event is hypothetically related to glutamate release after the ototoxic challenge that might directly activate resident cochlear macrophages in the sensory epithelium and protect the synaptic region from further injury in *CBA* mice [41,42]. Additionally, the G/F treatment induced the loss of ribbon synapses following OHC loss 15-day posttreatment in both mouse strains, with greater statistical significance in *B6* mice (Table 3).

*B6* and *CBA* mice are arguably the two most widely selected strains in auditory research. A review article revealed the number of studies reporting synaptic damage in *CBA* mice is 6 times more than that in *B6* mice [43], with cochlear synaptopathy more prevalent in *CBA* mice than *B6* mice [44]. There also appears to be a wide spectrum of ribbon synaptic vulnerability across species and among various mouse strains. Hypothetically, individuals with a robust OHC function and resistance to environmental insults are more prone to ribbon synaptic damage due to elevated cochlear amplification [45]. If this hypothesis holds true in the G/F-induced ototoxic situation, we would expect to see more synaptic damage in *CBA* mice, given that there was slightly less OHC loss 15-day posttreatment (though not significant). What we actually observed here is that the severity of OHC damage and synaptic damage were more likely synchronized to each other. While SGNs and ribbon synapses are not the primary ototoxic target of the G/F treatment, they are nonetheless targeted. This could provide rationalization against the cochlear amplification hypothesis. In addition, the slow clearance of gentamicin in the cochlea after the one-time administration is another rational basis for disrupting the inverse relationship between OHC survival and ribbon survival.

## 5. Conclusions

Combined 400 G/200 F treatment induced a multitude of rapid, overt cochlear damage responses with a substantial inter-animal variation. Two weeks after treatment, the cochlear damage was manifested by hair cell loss, ribbon synaptic damage, and macrophage activation in the BM. Notably, the G/F-activated BM macrophages were largely correlated to the severity of cochlear damage, particularly the OHC damage. Gauging either physiologically or morphologically, and compared to the *CBA* counterpart, *B6* cochleae were more vulnerable to the ototoxic challenge with slowly initiated but escalated macrophage activation. Additionally, the ribbon synaptic integrity was sustained considerably as the OHCs deteriorated in *CBA* mice. The innate and differential otoprotection observed in *CBA* mice appears to be associated to the prompt activation of cochlear macrophages, as well as a certain level of synaptogenesis after the combined G/F treatment. *In toto*, this study suggests that an agile response of innate immunity in the cochlea is otoprotective upon ototoxic insults while an elevated and sustained innate immune activity is implicated in the age-related hearing loss.

## Figures and Tables

**Table 1:** Ordinary two-way ANOVA statistics on 400 G/200 F-induced ABR threshold shifts. n.s. = not significant. * *p* < 0.05, **** *p* < 0.0001.

Comparison	*p* Value	*p* Value Summary	F (DFn, DFd)	n
Pre-treatment, *B6 vs CBA*	0.5456	n.s.	F (1, 300) = 0.3661	21 *vs* 31
2 d posttreatment, *B6 vs CBA*	0.0462	*	F (1, 312) = 4.006	30 *vs* 24
15 d posttreatment, *B6 vs CBA*	<0.0001	****	F (1, 282) = 39.49	26 *vs* 23
*B6*, pre- *vs* 2 d posttreatment	<0.0001	****	F (1, 294) = 105.6	21 *vs* 30
*CBA*, pre- *vs* 2 d posttreatment	<0.0001	****	F (1, 318) = 72.56	31 *vs* 24
*B6*, 2 d *vs* 15 d posttreatment	<0.0001	****	F (1, 324) = 43.97	30 *vs* 26
*CBA*, 2 d *vs* 15 d posttreatment	0.0453	*	F (1, 270) = 4.046	24 *vs* 23

**Table 2:** Ordinary two-way ANOVA statistics on 400 G/200 F-induced variation of ribbon density. n.s. = not significant. * *p* < 0.05, ** *p* < 0.01.

Comparison	*p* Value	*p* Value Summary	F (DFn, DFd)	n
*B6*, pre- *vs* 2 d posttreatment	0.0497	*	F (1, 57) = 4.020	5 *vs* 12
*B6*, pre- *vs* 15 d posttreatment	0.0048	**	F (1, 90) = 8.358	5 *vs* 20
*B6*, 2 d *vs* 15 d posttreatment	0.0027	**	F (1, 117) = 9.384	12 *vs* 20
*CBA*, pre- *vs* 2 d posttreatment	0.44	n.s.	F (1, 61) = 0.6041	6 *vs* 12
*CBA*, pre- *vs* 15 d posttreatment	0.0451	*	F (1, 65) = 4.175	6 *vs* 14
*CBA*, 2 d *vs* 15 d posttreatment	0.0123	*	F (1, 92) = 6.528	12 *vs* 14

**Table 3:** Ordinary two-way ANOVA statistics on 400 G/200 F-enhanced BM macrophage density. n.s. = not significant. * *p* < 0.05, ** *p* < 0.01, **** *p* < 0.0001.

Comparison	*p* Value	*p* Value Summary	F (DFn, DFd)	n
Pre-treatment, *B6 vs CBA*	0.0101	*	F (1, 35) = 7.396	5 *vs* 6
2 d posttreatment, *B6 vs CBA*	0.0015	**	F (1, 50) = 11.36	9 *vs* 6
15 d posttreatment, *B6 vs CBA*	0.0019	**	F (1, 76) = 10.30	14 *vs* 7
*B6*, pre- *vs* 2 d posttreatment	0.5306	n.s.	F (1, 46) = 0.3992	5 *vs* 9
*B6*, pre- *vs* 15 d posttreatment	<0.0001	****	F (1, 68) = 18.27	5 *vs* 14
*B6*, 2 d *vs* 15 d posttreatment	<0.0001	****	F (1, 82) = 35.76	9 *vs* 14
*CBA*, pre- *vs* 2 d posttreatment	<0.0001	****	F (1, 39) = 31.38	6 *vs* 6
*CBA*, pre- *vs* 15 d posttreatment	<0.0001	****	F (1, 43) = 28.85	6 *vs* 7
*CBA*, 2 d *vs* 15 d posttreatment	0.7597	n.s.	F (1, 44) = 0.09477	6 *vs* 7

## Data Availability

The data that support the findings of this study are inclusively provided within the present manuscript and supplementary material.

## Supplementary Materials

The following supporting information can be downloaded at: https://www.mdpi.com/article/10.3390/ijms23137343/s1.
