# Supplementary material for: Effects of Combined Gentamicin and Furosemide Treatment on Cochlear Macrophages
Source: Int J Mol Sci. 2022 Jul 1;23(13):7343. doi: 10.3390/ijms23137343 (PMC9266920; doi:10.3390/ijms23137343)
Supplement: Supplementary file 1 [file ijms-23-07343-s001.zip › Supplementary Materials.pdf]

Supplementary Materials

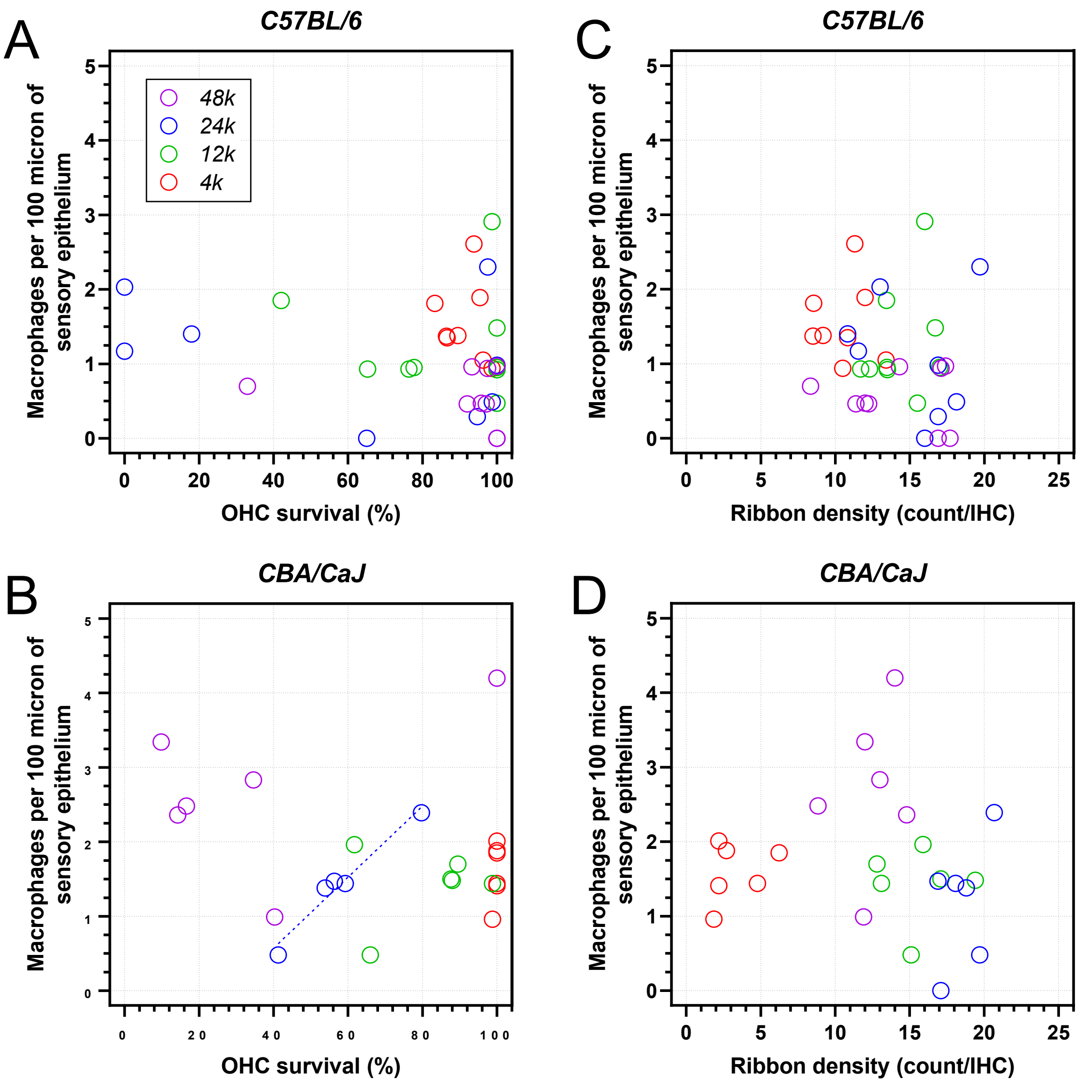

Supplemental Figure S1: Effect of 400G/200F treatment on ABR wave-I amplitudes at 90-, 85-, and 80-dB SPL to 12-kHz tones, in *B6* and *CBA* mice.

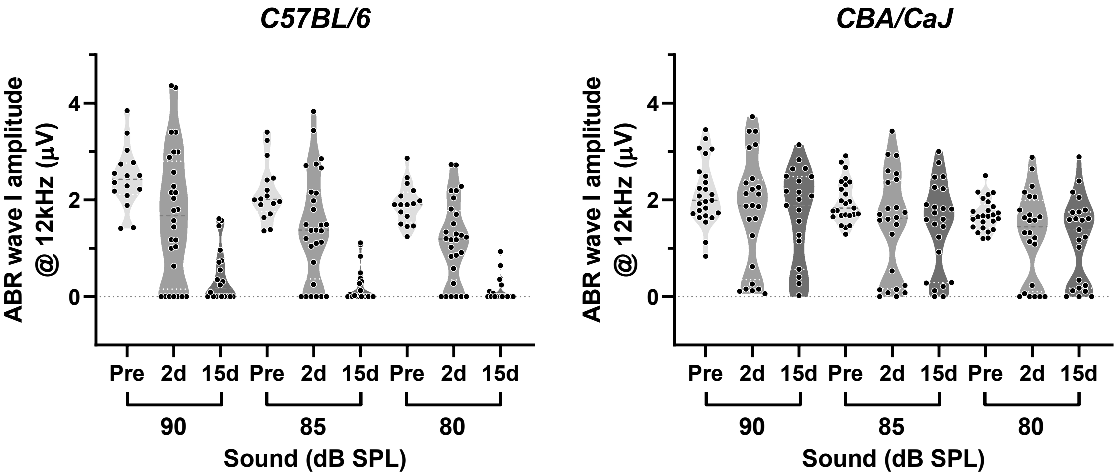

Supplemental Figure S2: Correlation between IHC ribbon density and ABR wave-I amplitude (90 dB SPL, 12 kHz) 2 and 15 days after 400G/200F treatment in *B6* and *CBA* mice. Red symbols depict an OHC survival rate ≥90%. Positive correlation was only observed in *B6* mice 2-day posttreatment.

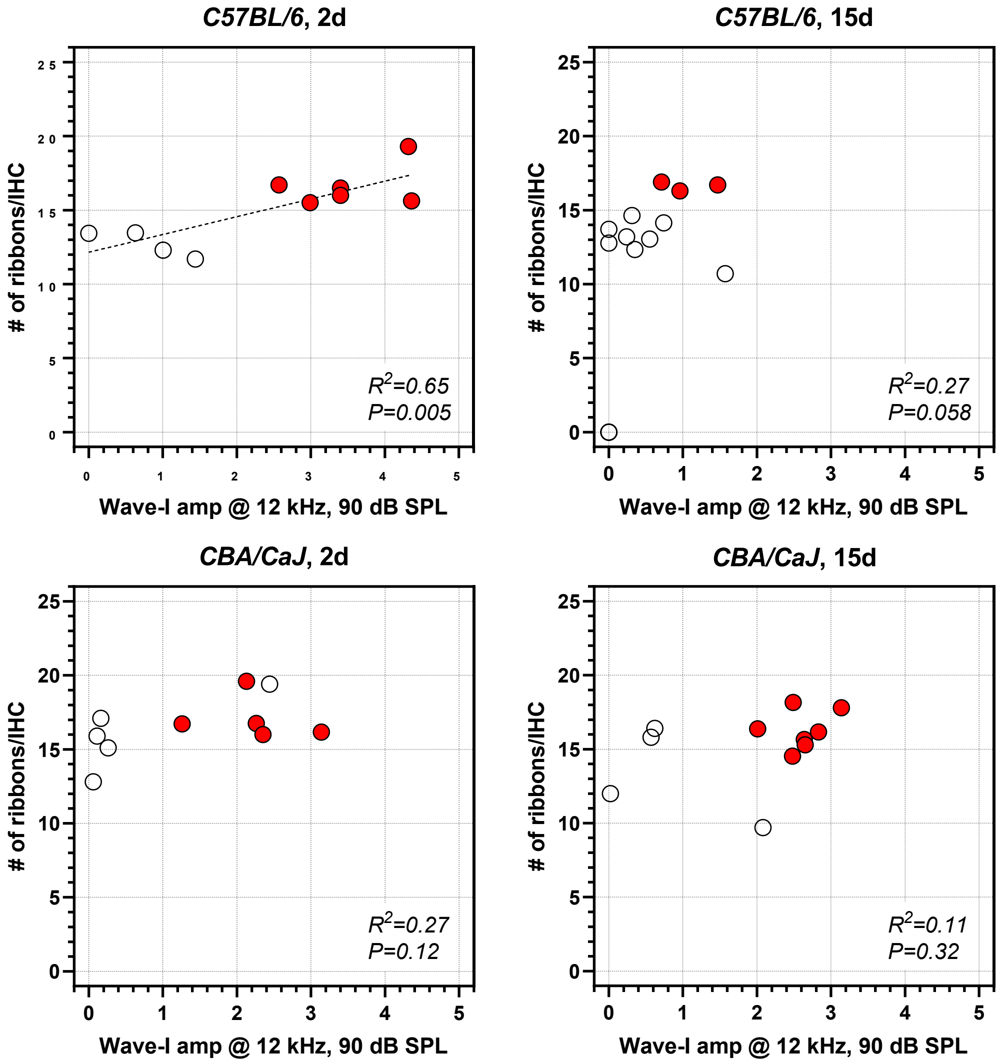

Supplemental Figure S3: Correlation between BM macrophage density and OHC survival at multiple cochlear locations 2 days after 400G/200F in *B6* and *CBA* mice. Positive correlation was only observed at the 24-kHz frequency location in *CBA* mice.
